# Effects of geometry and topography on Min-protein dynamics

**DOI:** 10.1371/journal.pone.0203050

**Published:** 2018-08-30

**Authors:** Lukas Wettmann, Mike Bonny, Karsten Kruse

**Affiliations:** 1 Theoretische Physik, Universität des Saarlandes, Saarbrücken, Germany; 2 NCCR Chemical Biology, Departments of Biochemistry and Theoretical Physics, University of Geneva, Geneva, Switzerland; University of California San Diego, UNITED STATES

## Abstract

In the rod-shaped bacterium *Escherichia coli*, the center is selected by the Min-proteins as the site of cell division. To this end, the proteins periodically translocate between the two cell poles, where they suppress assembly of the cell division machinery. Ample evidence notably obtained from *in vitro* reconstitution experiments suggests that the oscillatory pattern results from self-organization of the proteins MinD and MinE in presence of a membrane. A mechanism built on cooperative membrane attachment of MinD and persistent MinD removal from the membrane induced by MinE has been shown to be able to reproduce the observed Min-protein patterns in rod-shaped *E. coli* and on flat supported lipid bilayers. Here, we report our results of a numerical investigation of patterns generated by this mechanism in various geoemtries. Notably, we consider the dynamics on membrane patches of different forms, on topographically structured lipid bilayers, and in closed geometries of various shapes. We find that all previously described patterns can be reproduced by the mechanism. However, it requires different parameter sets for reproducing the patterns in closed and in open geometries.

## Introduction

The formation of static and dynamic patterns is one of the hallmarks of living systems. Different mechanisms have been identified to be instrumental in this context. The concept of self-organization [1] is particularly appealing, because it gives a comprehensive account of pattern formation and occasionally even suggests mechanisms of behavioral system responses to external stimuli. In its strict sense, self-organization refers to a process involving many “agents” of a few different kinds acting together to form structures that they would not be able to form in absence of their interactions. On a microscopic level, self-organization results in the simplest case from reactions between molecules and their transport through diffusion. In a seminal work, Turing showed that such systems can lead to stationary or dynamic structures if the diffusion constants of at least two molecular species differ sufficiently [2].

Although many biological patterns have been suggested to result from reaction diffusion systems [3], only a few specific examples have directly been shown to be Turing patterns [4]. The probably best studied example is the Min system in *Eschrichia coli* [5, 6]. There, the proteins MinD and MinE, that are involved in selecting the cell center as future site of division, oscillate between the two ends of the rod-shaped cells [7, 8]. In addition, standing waves with multiple nodes and traveling waves have been reported in long cells [7, 9]. As an interesting twist compared to Turing’s original suggestion, protein synthesis and degradation is not involved in Min-protein pattern formation and differences in the diffusion constants are generated by the proteins switching between a cytoplasmic and a membrane-bound state.

In brief, the molecular mechanisms underlying Min-protein pattern formation are as follows: MinD is an ATPase [10], that is, it can bind Adenosine triphosphate (ATP) and hydrolyze it. After binding ATP, MinD experiences a conformational change, which increases MinD’s affinity for binding to a lipid bilayer [11–15]. It can then recruit MinE to the membrane, which in turn increases the ATPase activity of MinD and in this way drives it off the membrane. In a normal-sized *E. coli* cell, MinD that has assembled at one pole, recruits MinE. MinE then induces detachment of MinD, which subsequently assembles at the opposite pole. This process repeats itself with a period of roughly a minute.

Theoretical analysis has played a key role in the analysis of the Min system. Early studies promoted the idea that the Min-protein patterns do not depend on cellular cues, but emerge from self-organization [16–19]. Furthermore, theoretical analysis suggested that MinD and MinE should be able to form patterns in vitro on a flat membrane [18, 20]. This prediction was later confirmed experimentally [21, 22]. Theory was also instrumental in showing that the same mechanism underlying pattern formation *in vitro* can also explain the patterns observed *in vivo* [9]. Furthermore, it predicted the existence of traveling waves, which were subsequently found experimentally [9]. Meanwhile, standing waves similar to the patterns *in vivo* have been also obtained *in vitro* [23–25] further supporting that a common mechanism underlies the patterns formed *in vitro* and in living bacteria.

In studying the mechanism underlying Min-protein pattern formation, changes in geometry proved to be a most insightful tool. In a bacterial context, length [7, 26], width [9, 27], and form [27, 28] were found to qualitatively change the patterns, whereas *in vitro*, restriction of protein binding to membrane patches of various forms [29] and topographical surface structures [23, 30] strongly influenced the pattern. Furthermore, patterns in different micro-fabricated compartments depend strongly on the geometry [25]. Since most of the kinetic parameters governing Min-protein dynamics are still unknown [31], the responses of the patterns to changes in geometry provide to date the strongest constraints on possible mechanism.

The currently most successful effective description of the Min-protein dynamics includes co-operative attachment of MinD and persistent removal of MinD by MinE [9]. It has been shown to yield all patterns that have been reported in living *E. coli* for various lengths and protein concentrations and also describes the traveling waves observed *in vitro*, although different parameter sets have to be used for describing *in vitro* and *in vivo* situations. Less attention has been paid to the theoretical analysis of Min-protein patterns in “unconventional” geometries, including Y-shaped [32], triangular- or circular-shaped [27], and giant *E. coli* [9, 27], as well as membrane patches on flat surfaces [29] and to topographically structured surfaces *in vitro* [23, 30]. In the present work, we show that contrary to claims made elsewhere [33], the description introduced in Refs. [9, 29] is able to account also for the Min-protein patterns in these unconventional geometries. We start by recalling the corresponding dynamic equations presented in Ref. [9]. We then numerically analyze the dynamic equations for *in vitro* geometries and proceed to bacterial geometries. Finally, we discuss possible prospects for further theoretical studies.

## Materials and methods

### Numerical solutions of the dynamic equations

We solved the dynamic Eqs (1)–(7) in the *in vitro* as well as in the *in vivo* geometries by using Comsol Multiphysics 5.0, which is a solver for partial differential equations based on the finite element method (FEM). For the calculations in closed geometries, the maximal grid size was 1.5*μ*m. The finite element mesh was generated automatically by Comsol and we only specified the maximal grid size. The mesh consisted of tetrahedra in the bulk and correspondingly triangles on the membrane. For the computations in open geometries, we had to use a separate finite element mesh with a finer grid size on the membrane. We checked that the obtained patterns did not change for meshes with smaller maximal grid size, S1 and S2 Movies. For the calculations in open geometries, we used a maximal grid size of 2*μ*m in the surface domain and of 15*μ*m in the buffer domain. Specific values used in the calculations are given in the corresponding figure captions. As initial condition, we used homogenous distributions of cytosolic proteins with a random perturbation of 5–10%. The initial surface densities were chosen to be zero for the closed geometries. For the calculations in the open geometries, the initial surface densities were different from zero in a semi-annulus to rapidly induce a spiral.

## Results

### Min-protein dynamics

**Fig 1:**
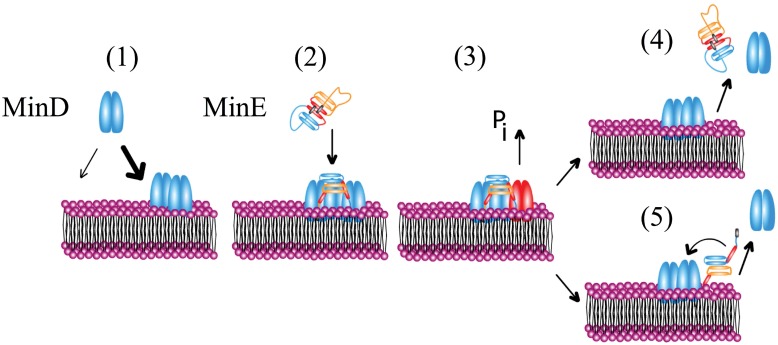
Illustration of the molecular processes involved in Min-protein dynamics. 1) Co-operative binding of MinD to a lipid bilayer. 2) Recruitment of MinE to the bilayer. 3) ATP hydrolysis. 4) Simultaneous detachment of MinD and MinE. 5) Detachment of MinD while MinE rests on the membrane and possibly reforms a complex with MinD.

In this work, we use the model introduced in Ref [9] and follow the presentation of the dynamic equations given there. The model accounts for the following molecular processes, Fig 1: MinD binds to the lipid bilayer. For MinD proteins, this process involves several steps, as binding of ATP leads to the formation of an amphipathic helix at the C-terminus giving the protein an increased affinity for binding lipids bilayers [11–15]. In addition, MinD dimerizes after binding ATP. Only in this form it translocates to the cytoplasmic membrane. The binding kinetics of MinD to the membrane differs from Langmuir kinetics indicating significant cooperative effects during the binding process [13, 34, 35]. Experiments *in vitro* on vesicles incubated in a buffer containing MinD suggest a two-step process of MinD binding: first it binds to the membrane and subsequently forms clusters [36]. Some theoretical works have considered several substeps in membrane binding [18, 37, 38], but more work is necessary to clearly identify the origin of deviations form Langmuir kinetics. We thus follow Ref. [19] and describe MinD binding to the membrane by an effectively non-linear process such that membrane-bound MinD favours further binding of MinD.

MinE is recruited to the cytoplasmic membrane by membrane-bound MinD dimers through binding to a site located at the MinD-dimer interface [15, 39, 40]. At the same time, MinE interacts directly with the membrane through an amphipathic *α*-helix [41] following a dramatic conformational change induced by an interaction with MinD [42]. The binding of MinE stimulates the ATPase activity of MinD and in this way triggers MinD detachment from the membrane [12, 13]. Because of its direct interaction with the membrane, MinE can remain on the membrane after MinD detachment for a short period during which it can associate with another membrane-bound MinD dimer [22, 41]. This process has been considered previously in a stochastic description of Min-protein dynamics in cellular geometries [43].

To keep the equations simple, we only consider MinD dimers and do not distinguish between the states when ATP is bound or not. In our meanfield description, the volume densities of MinD and MinE not bound to the membrane are denoted by *c*_*D*_ and *c*_*E*_, respectively. The surface densities of membrane-bound MinD, MinE, and MinDE complexes are denoted by *c*_*d*_, *c*_*e*_, and *c*_*de*_, respectively, and are defined only on the surfaces representing the membrane. The time evolution of these densities is governed by the following dynamic equations

$$\begin{array}{ccc} \partial_{t}c_{D} & = & D_{D}\Delta c_{D} \end{array}$$ (1)

$$\begin{array}{ccc} \partial_{t}c_{E} & = & D_{E}\Delta c_{E} \end{array}$$ (2)

$$\begin{array}{ccc} \partial_{t}c_{d} & = & D_{d}\Delta_{\|}c_{d}+c_{D}\left(\omega_{D}+\omega_{dD}c_{d}\right)\left(c_{\text{max}}-c_{d}-c_{de}\right)/c_{\text{max}}\\ & & -\omega_{E}c_{E}c_{d}-\omega_{ed}c_{e}c_{d} \end{array}$$ (3)

$$\begin{array}{ccc} \partial_{t}c_{de} & = & D_{de}\Delta_{\|}c_{de}+\omega_{E}c_{E}c_{d}+\omega_{ed}c_{e}c_{d}\\ & & -\left(\omega_{de,m}+\omega_{de,c}\right)c_{de} \end{array}$$ (4)

$$\begin{array}{ccc} \partial_{t}c_{e} & = & D_{e}\Delta_{\|}c_{e}+\omega_{de,m}c_{de}-\omega_{ed}c_{e}c_{d}-\omega_{e}c_{e}. \end{array}$$ (5)

In Eqs (3)–(5), Δ_‖_ denotes the Laplace-operator on the surface and *D*_*d*_, *D*_*e*_, and *D*_*de*_ are the respective diffusion constants of membrane-bound MinD, MinE, and MinDE. Furthermore, *c*_max_ is the maximal MinD density on the membrane. For Min-protein pattern formation on the membrane, it seems essential to limit the concentration of MinD on the membrane. For example, experiments *in vitro* suggest that cytosolic MinD needs to be depleted near the membrane [44] and the finite exchange rate of ADP for ATP on cytosolic MinD as introduced in Ref. [19] effectively has the same effect. For the *in vitro* geometries, we use a value of *c*_max_ that is close to the maximal packing density of MinD on the membrane, for the *in vivo* geometries we account for the presence of other membrane-bound molecules by using a roughly 5 times lower maximal density. In Eqs (3) and (4), the densities *c*_*D*_ and *c*_*E*_ are evaluated at the same points as the surface densities. In Eqs (1) and (2), Δ denotes the Laplace-operator in three dimensions and *D*_*D*_ and *D*_*E*_ are the diffusion constants for cytosolic MinD and MinE, respectively.

The remaining terms describe attachment of proteins to and their detachment from the lipid bilayer. MinD in the vicinity of the membrane binds at rate *ω*_*D*_ to the lipid bilayer. We capture cooperative effects in the binding process through increasing the binding rate by *ω*_*dD*_ times the local density of membrane-bound MinD. The formation of MinDE complexes by binding of MinE to membrane-bound MinD occurs at rate *ω*_*E*_*c*_*d*_. A MinDE complex can dissociate in two ways: either, both, MinD and MinE, detach from the membrane or only MinD leaves the membrane, whereas MinE stays on the lipid bilayer. The two processes occur at respective rates *ω*_*de*,*c*_ and *ω*_*de*,*m*_. Individual MinE dimers on the membrane associate with nearby membrane-bound MinD at rate *ω*_*ed*_*c*_*d*_ or dissociate from the membrane at rate *ω*_*e*_.

The dynamic equations for cytosolic MinD and MinE are complemented by boundary conditions on the diffusion currents that account for protein binding to and detachment from the membrane: The components of these currents orthogonal to the membrane equal the net attachment rate. Formally, we have

$$\begin{array}{cc} -D_{D}\nabla_{⊥}c_{D} & =c_{D}\left(\omega_{D}+\omega_{dD}c_{d}\right)\left(c_{\text{max}}-c_{d}-c_{de}\right)/c_{\text{max}}-\left(\omega_{de,m}+\omega_{de,c}\right)c_{de} \end{array}$$ (6)

$$\begin{array}{cc} -D_{E}\nabla_{⊥}c_{E} & =\omega_{E}c_{E}c_{d}-\omega_{e}c_{e}-\omega_{de,c}c_{de}. \end{array}$$ (7)

Here, ∇_⊥_ denotes the outward gradient normal to the boundary. Note, that Eqs (1)–(7) conserve the total protein number.

Various other mechanisms have been considered to describe Min-protein dynamics [45], but so far we lack sufficient molecular information to clearly favor one over the others. A comprehensive comparison with published experimental data is lacking for most models. For the dynamic Eqs (1)–(7) it has been shown that they produce the patterns observed in “standard” geometries [9]. Here, we show that also the patterns in unconventional geometries are captured by this system.

### Min-protein patterns in open geometries

It has been shown previously that the dynamic Eqs (1)–(7) reproduce essential features of Min-protein patterns in bacteria as well as in reconstitution experiments *in vitro* [9]. In Ref. [29], a two-dimensionsal version of these equations was employed to study Min-protein dynamics on patterned surfaces, see SI. It is currently unknown under which conditions it is possible to formally pass from the equations in three to those in two dimensions. Still for an appropriate choice of parameters, the 2d equations reproduce the patterns obtained from the 3d equations. In this section, we will show this explicitly for unstructured flat surfaces. We will then turn to structured surfaces, where we will restrict ourselves to calculations in 3d.

#### Comparison between 2d and 3d calculations

**Fig 2:**
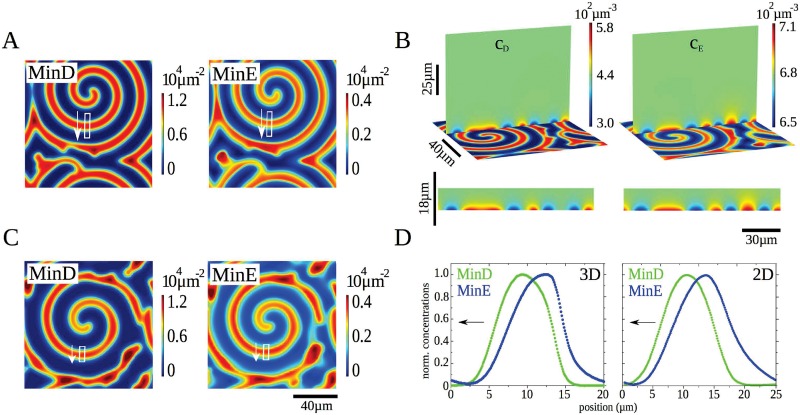
Min-protein dynamics in an open geometry. A) Densities of membrane-bound MinD and MinE, *c*_*d*_ + *c*_*de*_ and *c*_*de*_ + *c*_*e*_, respectively, from a calculation in 3d. B) Buffer densities of MinD and MinE from a calculation in 3d for the same time point shown in (A). C) Densities of membrane-bound MinD and MinE from a calculation in 2d. D) Density profiles of membrane-bound MinD and MinE in the white rectangles indicated in (A) and (C). Lateral extensions are 120 *μ*m × 120 *μ*m. In (A) and (B) the chamber height is 90 *μ*m. Other parameter values as in Table 1. The maximal grid size used in the computations was 2 *μ*m on the membrane and 15 *μ*m in the bulk.

Consider a system *in vitro* with a flat membrane and a lateral extension of 120 *μ*m times 120 *μ*m. For the 3d calculations the height is 90 *μ*m. We impose no-flux boundary condition on the top and periodic boundary conditions in the lateral directions. The parameters used in the following are given in Table 1. Solutions to the dynamic Eqs (1)–(7) and their two-dimensional counterparts on the membrane are shown in Fig 2. In both cases, we observe spirals with similar extensions, rotation frequencies, and wave speeds. Explicitly, the waves propagate at a velocity of about 1 *μ*m/s and have a wavelength of roughly 20 *μ*m, which are on the order of the speed and wavelength found *in vitro* [21]. The 3d calculations show that the pattern extends roughly 5 *μ*m into the bulk underlining the quasi-2d character of the pattern, Fig 2C.

**Table 1:** Parameter values used for the numerical solutions of the deterministic dynamic Eqs (1)–(7) in 3d and of Eqs (S1)-(S5) in 2d.

	*in vivo*	*in vitro (3d)*	*in vitro* (2d)
*D*_*D*_	$15\frac{\mu m^{2}}{s}$	$50\frac{\mu m^{2}}{s}$	$50\frac{\mu m^{2}}{s}$
*D*_*E*_	$12.5\frac{\mu m^{2}}{s}$	$50\frac{\mu m^{2}}{s}$	$50\frac{\mu m^{2}}{s}$
*D*_*d*_	$0.0125\frac{\mu m^{2}}{s}$	$0.3\frac{\mu m^{2}}{s}$	$0.24\frac{\mu m^{2}}{s}$
*D*_*e*_	$0.075\frac{\mu m^{2}}{s}$	$1.8\frac{\mu m^{2}}{s}$	$0.48\frac{\mu m^{2}}{s}$
*D*_*de*_	$0.0125\frac{\mu m^{2}}{s}$	$0.3\frac{\mu m^{2}}{s}$	$0.24\frac{\mu m^{2}}{s}$
*c*_max_	$5.4\cdot 10^{3}\;\frac{1}{\mu m^{2}}$	$2.75\cdot 10^{4}\;\frac{1}{\mu m^{2}}$	$2\cdot 10^{4}\;\frac{1}{\mu m^{2}}$
*ω*_*D*_	$0.025\frac{\mu m}{s}$	$5\cdot 10^{-4}\;\frac{\mu m}{s}$	$0.045\frac{1}{s}$
*ω*_*dD*_	$5\cdot 10^{-4}\;\frac{\mu m^{3}}{s}$	$3.18\cdot 10^{-3}\;\frac{\mu m^{3}}{s}$	$9\cdot 10^{-4}\;\frac{\mu m^{2}}{s}$
*ω*_*E*_	$5.2\cdot 10^{-5}\;\frac{\mu m^{3}}{s}$	$1.36\cdot 10^{-4}\;\frac{\mu m^{3}}{s}$	$4\cdot 10^{-4}\;\frac{\mu m^{2}}{s}$
*ω*_*ed*_	$0.174\frac{\mu m^{2}}{s}$	$4.9\cdot 10^{-3}\;\frac{\mu m^{2}}{s}$	$2.5\cdot 10^{-3}\;\frac{\mu m^{2}}{s}$
*ω*_*de*,*c*_	$0.02\frac{1}{s}$	$0.16\frac{1}{s}$	$0.08\frac{1}{s}$
*ω*_*de*,*m*_	$0.375\frac{1}{s}$	$2.52\frac{1}{s}$	$0.8\frac{1}{s}$
*ω*_*e*_	$0.125\frac{1}{s}$	$0.5\frac{1}{s}$	$0.08\frac{1}{s}$

The total MinD and MinE concentrations, *C*_*D*_ and *C*_*E*_, and the system length varied between simulations and are given in the corresponding figure captions.

The wave profiles in 2d and 3d, Fig 2D, present similar features as their experimental counterparts obtained *in vitro* [22]: the increase in MinD precedes that in MinE, the maximal MinE concentration is reached after the maximum in MinD was attained, and the profiles are asymmetric with respect to their maxima: the decrease is sharper than the increase. These profiles do not present a very sharp maximum followed by a rapid decrease in the MinE density at the trailing edge that is observed *in vitro* [22] and which parallels the MinE ring observed on *E. coli* [46]. The ability of a single MinE molecule to induce detachment of several MinD dimers in a row can produce this feature [38, 43] and indeed there are parameter sets for which Eqs (1)–(7) reproduce this feature. However, these parameter sets reproduce less well other features of the system. We refrained from an extended search in parameter space and from modifying the non-linear terms in the dynamic equations to improve the match between experimental and theoretical profiles. In view of the high computational costs involved and our current limited knowledge about the molecular details underlying the Min-protein dynamics this seems to be premature. Instead, our more modest goal here is to show that the mechanism presented above provides a unifying framework for the patterns observed in various geometries.

**Fig 3:**
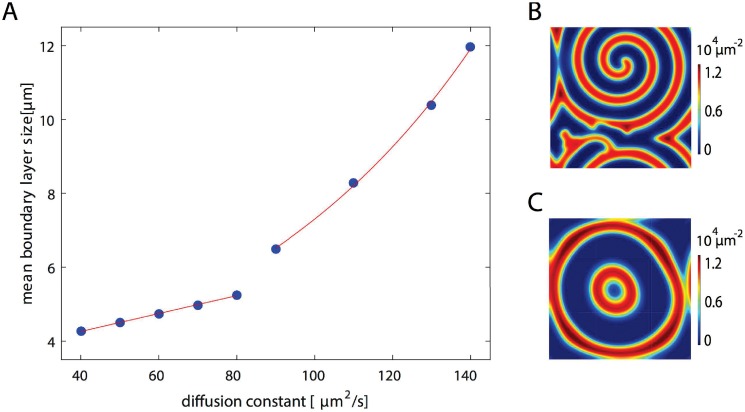
Boundary layer size in dependence of cytosolic diffusion constant. A) Mean boundary layer size as a function of the diffusion constant *D* ≡ *D*_*D*_ = *D*_*E*_. Red lines represent a linear and an exponential fit to the data points for *D* < 90 *μ*m^2^/s and for *D* > 90 *μ*m^2^/s, respectively. B, C) Density of membrane-bound MinD from a solution for *D* = 50 *μ*m^2^/s (B) and for *D* = 130 *μ*m^2^/s (C). Other parameter values as in Fig 2. The maximal grid size used in the computations was 2 *μ*m on the membrane and 15 *μ*m in the bulk.

To further compare the 2d and 3d patterns, we investigated the thickness of the boundary layer as a function of the bulk diffusion constants *D*_*D*_ and *D*_*E*_, where we choose *D*_*D*_ = *D*_*E*_, Fig 3A. The protein densities exponentially approach the bulk value as the distance to the membrane increases. We define the boundary layer thickness as the characteristic length of the exponential, where we have averaged values from various simulations with different initial conditions and at different locations. For diffusion constants below 80 *μ*m^2^/s, the thickness of the boundary layer increases linearly with the diffusion constant. Afterwards the increase is super linear. One has to note, however, that the pattern changes qualitatively for a critical diffusion constant of 90 *μ*m^2^/s. Below this value, the proteins organize in a spiral, Fig 3B, above they form expanding concentric rings, Fig 3C. These observations suggest that the patterns in 3d and in 2d should resemble each other most for small diffusion constants. However, for diffusion constants below 40 *μ*m^2^/s, the homogenous state is stable and no patterns are formed.

#### Patterned surfaces

**Fig 4:**
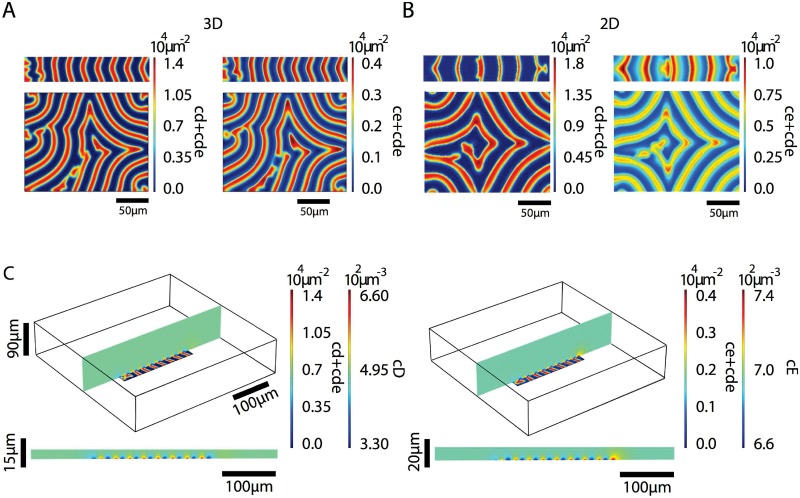
Patterns on rectangular membrane patches. A, B) Densities of membrane-bound MinD (left) and MinE (right) from calculations in 3d (A) and 2d (B). Top: size 170 *μ*m × 35 *μ*m, bottom: size 170 *μ*m × 135 *μ*m. C) Distribution of unbound MinD (left) and MinE (right) in a plane perpendicular to the patch. Parameter values as in Table 1. The maximal grid size used in the computations was 2 *μ*m on the membrane and 15 *μ*m in the bulk.

Min-protein surface waves have been found to be guided by geometrical constraints [29]. For rectangular membrane patches, the waves align with the rectangle’s long axis if the short axis is shorter than their wave length. If the short axis is larger, then the pattern shows spiral defects as in the case of (essentially) unbounded membranes [29]. The solutions to the dynamic Eqs (1)–(7) show the same behavior, Fig 4. Here, we have used reflecting boundary conditions on the diffusion current in the domains outside the membrane patches. Note that for the parameters chosen, the pattern is independent of the size of the domain surrounding the rectangle, where MinD and MinE cannot bind. Indeed, the patterns extend only a few *μ*m in lateral direction outside the membrane patch.

**Fig 5:**
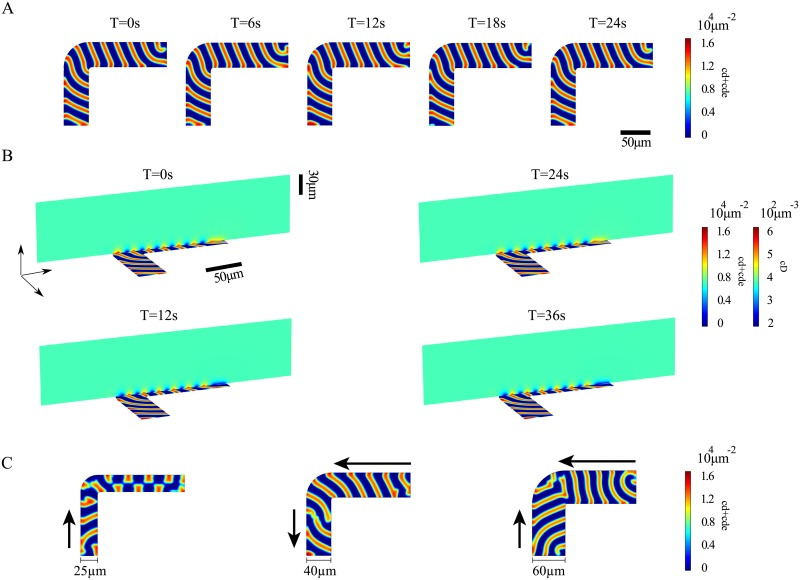
Patterns on L-shaped membrane patches. Results from calculations in 3d. A) Membrane densities of MinD at different time points. B) Bulk and membrane densities of MinD at different time points. C) Snapshots of the MinD density on L-shaped membrane patches of different thickness. The arrows indicate the propagation direction of the traveling waves. On the long arm of the patch of thickness 25 *μ*m a standing wave is formed. Parameter values as in Table 1. The maximal grid size used in the computations was 2 *μ*m on the membrane and 15 *μ*m in the bulk.

Why does the direction of wave propagation align with the long axis of a rectangle that is narrow enough? While we will not give a detailed analysis here, we can give some qualitative insight into this phenomenon. Consider the front of a Min protein wave that reaches a straight edge of a patch. At the edge the concentration of membrane-bound MinE will increase, because their diffusion on the membrane is constrained by the edge. As a consequence, wave propagation will accelerate near the edge and the wave front will gradually bend from its bulk orientation to form a right angle with the edge [29], notably Fig 5A–5C. If two parallel edges are close enough, in our case around 40 *μ*m, then the orientation of the wave front is determined by this boundary effect. This effect is best studied using an L-shaped domain, Fig 5 and S3 Movie. In this case, the wave follows the turn, which requires the wave to be faster at the outer boundary compared to the inner boundary of the turn. This behavior is in agreement with experimental observations [29].

Let us note that guiding of the wave is lost if the width is too small. A reduction of the width below roughly 30 *μ*m leads to a change of the pattern. On the longer arm of the patch a kind of standing wave is formed in this case, whereas a traveling wave is present on the shorter arm, Fig 5C and S4 Movie. When increasing the width, the pattern also changes, Fig 5C and S5 Movie. This is expected as the boundary effects are in this case less important for the pattern. For a width of 60 *μ*m, spiral cores form at two opposite corners of the patch and on both arms waves travel towards the bend.

#### Topographically structured surfaces

**Fig 6:**
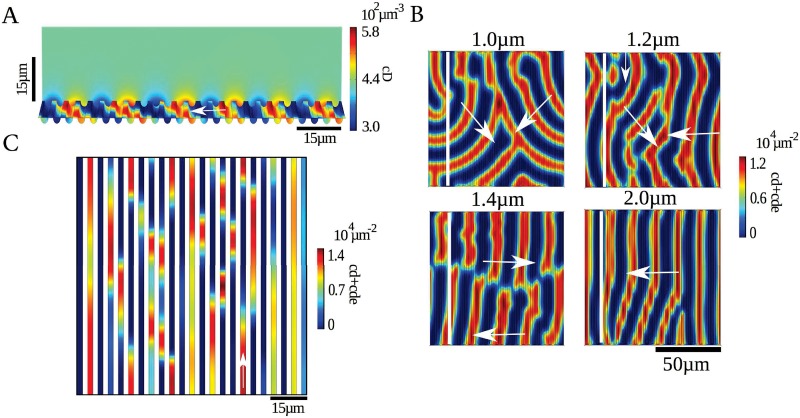
Patterns on topographically structured membranes. Results from calculations in 3d. A) Bulk density *c*_*D*_ of MinD for grooves separated by 2.2 *μ*m with a width of 2.1 *μ*m and a depth of 1.2 *μ*m. B) Surface density *c*_*d*_ + *c*_*de*_ of MinD for different groove widths. The white lines indicate the orientation of the grooves, the arrows indicate the direction of wave propagation. The grooves are separated by 2.2 *μ*m, have a width of 2.1 *μ*m, and their depth is indicated on the panels. Parameter values as in Table 1. C) Patterns on patches of width 2.2 *μ*m and distance 2.1 *μ*m between patches. Arrows indicate the direction of wave propagation. The maximal grid size used in the computations was 2 *μ*m on the membrane and 15 *μ*m in the bulk.

In addition to lateral boundaries, topological surface structures have been reported to guide Min-protein waves [30]. In particular, on a parallel grating with grooves of 8 *μ*m height and 5 *μ*m width, planar Min protein waves were observed to move perpendicular to the orientation of the grooves. Solutions to Eqs (1)–(7) in 3d show the same property as soon as the depth of the grooves exceeds a critical value, Fig 6A and 6B and S6 Movie. Here, the reason is that the Min-protein concentrations are increased within the grooves, which speeds up the waves. If a planar wave front hits a groove at an angle, the lagging parts of the wave front will be accelerated and thus lead to a turning of the wave front towards the groove. This is in striking contrast to the patterns on striped patches with a width equal to the separation of the grooves and a distance equal to the width of the grooves: in this case, waves propagate along the patches, that is, in the direction perpendicular to that of waves propagating on the grating, Fig 6C.

### Min-protein dynamics in closed geometries

For closed geometries like wild-type, rod-shaped *E coli* cells, the best known patterns of the Min proteins are pole-to-pole oscillations. In addition, the Min proteins can form standing waves with several nodes [7], switch stochastically between the cell poles [26, 47], and organize into traveling waves [9]. These patterns emerge in longer than normal cells and/or upon over-expression of the Min proteins. We have already shown in earlier work that the dynamic Eqs (1)–(7) are able to reproduce these patterns [9]. We will now show that they are able to reproduce the patterns in closed geometries of different shapes that have been studies experimentally.

#### Elementary geometric bodies

*Escherichia coli* can be forced to acquire aberrant shapes by growing it in appropriate microstructures [48]. This method has been used to study the Min-protein patterns in deformed *E. coli* [27]. The bacteria laterally adopted shapes of elementary geometric bodies like triangles, rectangles, and circles, while keeping an essentially constant thickness of about 1 *μ*m. In these geometries with a maximal lateral extension of 5 *μ*m, the proteins formed standing waves along one of the geometry’s symmetry axes [27]. For example, in an equilateral triangular shape, the proteins oscillate between one corner and the opposing side, which were chosen spontaneously among the three possibilities. Similarly, in a disk the rotational symmetry was spontaneously broken and a standing wave formed that aligned with a straight line through the disk’s centre.

**Fig 7:**
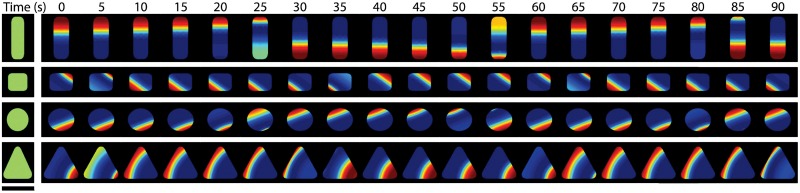
Oscillations in elementary geometric bodies. Results from calculations in 3d. Scale bar: 5 *μ*m. The height of the domain was 1.1 *μ*m. The average concentrations were *C*_*D*0_ = 9 ⋅ 10^2^ *μ*m^−3^ and *C*_*E*0_ = 9 ⋅ 10^2^ *μ*m^−3^. Other parameter values as in Table 1. The maximal grid size used in the computations was 0.5 *μ*m.

We solved the dynamic Eqs (1)–(7) in the same geometries used in Ref. [27]. As shown in Fig 7, for each of the elementary geometric bodies (rectangle, square, disk, triangle) stable pole-to-pole oscillations occur along a fixed axis that coincides with one of the body’s symmetry axes (S7–S10 Movies). More specifically, the selected symmetry axis is among those that are the longest for a given shape. Note, that the temporal period is essentially independent of the geometry. Instead it is determined by the extension of the standing wave’s axis. In agreement with the experiments, the patterns were identical on the bottom and the top of the forms.

**Fig 8:**
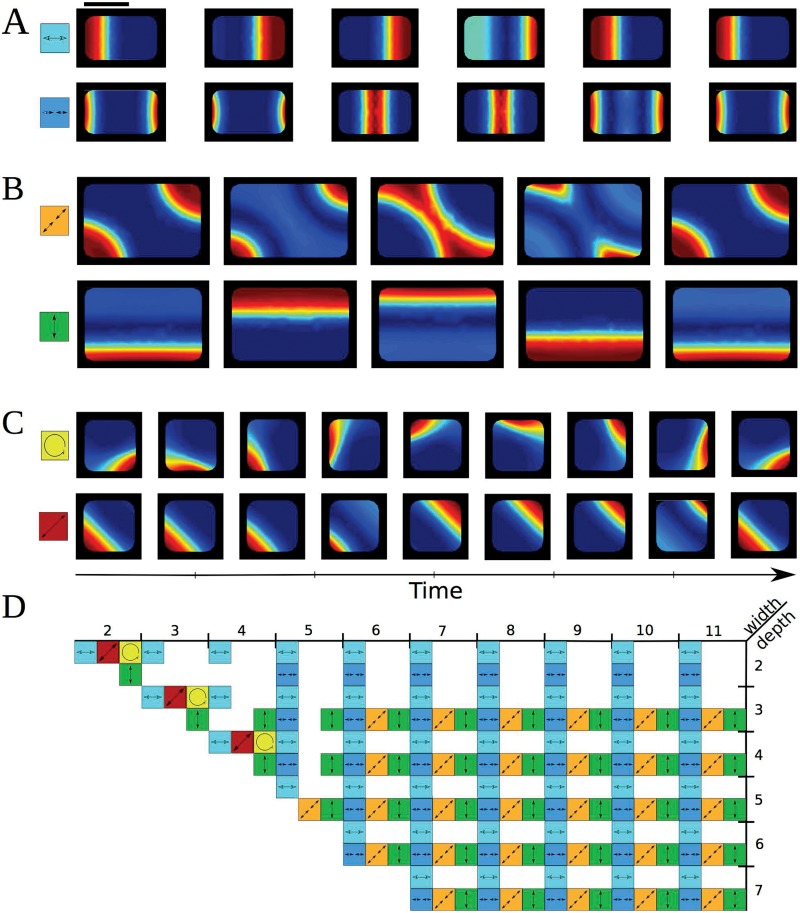
Examples of possible oscillation patterns found in rectangular cells. A) Standing waves with one (top) and two (bottom) nodes in a rectangle of size 3 *μ*m × 5 *μ*m. Scale bar: 3 *μ*m. B) Standing waves along the diagonal and along the short axis in a rectangle of size 5 *μ*m × 8 *μ*m. Same scale as in (A). C) Traveling wave around the perimeter and standing wave along the diagonal of a square of size 3 *μ*m × 3 *μ*m. Same scale as in (A). D) Summary of all patterns observed in rectangular geometries of various sizes. Results from calculations in 3d. Parameter values as in Fig 7. The maximal grid size used in the computations was 0.5 *μ*m (A), 1.1 *μ*m (B) and 0.45 *μ*m (C).

Several patterns can coexist. Coexistence of several Min-protein patterns has been reported before for the dynamic equations introduced in Ref. [49], see [50, 51]. All of the patterns can be decomposed into standing wave patterns along the rectangles’ symmetry axes with appropriate relative phases. As the lateral extensions of the domains were increased new stable patterns appeared, Fig 8 and S11–S14 Movies. Similar to the experiments in Ref. [27], we found stable standing waves with one node and with two nodes along the long axis of a rectangle of size 3 *μ*m × 5 *μ*m, Fig 8A. In larger rectangles of size 5 *μ*m × 8 *μ*m, additional stable standing waves along the diagonal and along the short axis could be observed, Fig 8B. Along the diagonal, the standing wave has two nodes. In square domains, also waves traveling along the domain’s perimeter exist, Fig 8C. Again, all these patterns can be decomposed into standing waves along the two diagonals with an appropriate phase. Given that the dynamic equations are non-linear, this feature is rather remarkable. The system chooses spontaneously one of the stable patterns depending on the initial conditions. To obtain the symmetric patterns shown in Fig 7, we chose the initial distribution of proteins to be inhomogeneous along one of the symmetry axes. In presence of molecular noise, one expects stochastic switches between the different stable patterns [51] similar to the stochastic switching between the two cell poles in short bacteria with over-expressed Min proteins [52].

A comprehensive presentation of the possible patterns in rectangular systems of different sizes and different aspect ratios is shown in Fig 8D. Waves traveling around the perimeter are restricted to square patterns. As long as the shorter side of the rectangle is 2 *μ*m in length, there are no standing waves along this direction unless the geometry is square.

#### Branched bodies

The deletion of certain penicillin binding proteins causes *E. coli* cells to keep their cylindrical shape but develop new cell poles by branching [32]. In particular, Min-protein patterns in three-poled cells have been reported. In these geometries, the Min oscillations can persist, switching periodically between one and the other two branches. In addition, rotating patterns, where the Min proteins visit the different branches sequentially, have been observed. Using a stochastic version of the model presented in Ref. [19], these patterns were found as a function of the relative branch lengths [32]: In symmetric bacteria, symmetric standing waves have been found, whereas in geometries with the branches of different sizes, the rotating pattern emerged. In case, two of the branches were much longer than the third, pole-to-pole oscillations along the two long branches appeared.

**Fig 9:**
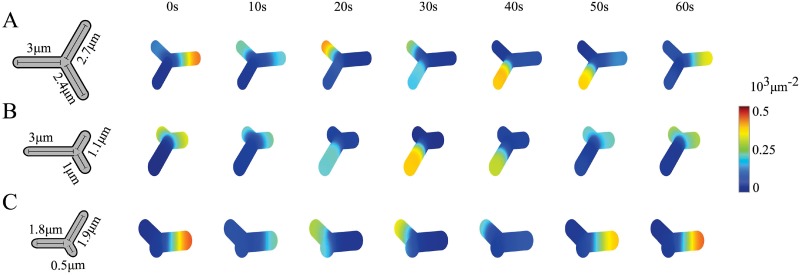
Oscillation patterns found in branched cells. A) Rotating wave pattern for branches of lengths 2.4 *μ*m, 2.7 *μ*m, and 3.0 *μ*m. B) Symmetric standing wave for branches of lengths 3.0 *μ*m, 1.1 *μ*m, and 1.0 *μ*m. C) Oscillation between two poles for branches of lengths 1.8 *μ*m, 1.9 *μ*m, and 0.5 *μ*m. Results from calculations in 3d. Parameter values as in Table 1. The maximal grid size used in the computations was 0.38 *μ*m.

The dynamic Eqs (1)–(7) yield similar patterns in this geometry, Fig 9 and S15–S17 Movies. We chose the angle between two branches to be 2*π*/3 and varied the lengths of the branches. As for the rod-shaped geometry, the distance between the tips of at least two branches needs to exceed a certain critical length for the system to produce oscillations. In the case that all branches are of a similar length, a rotational pattern, where high concentrations form subsequently in a clockwise or counter clockwise manner in the different branches emerges, Fig 9A. If one branch is much longer than the two others, a standing wave along the long branch forms, Fig 9B. Finally, if two branches are significantly longer than the third branch, a standing wave along these two branches forms, Fig 9C. The value of the critical length as well as the extent by which one or two branches needs to be longer than the remaining branch(es) to produce a standing wave rather than a rotational pattern depend on the values of the other parameters. In agreement with previous results, the number of nodes of the standing waves in one branch increases with its length (not shown).

#### Irregularly shaped bodies

**Fig 10:**
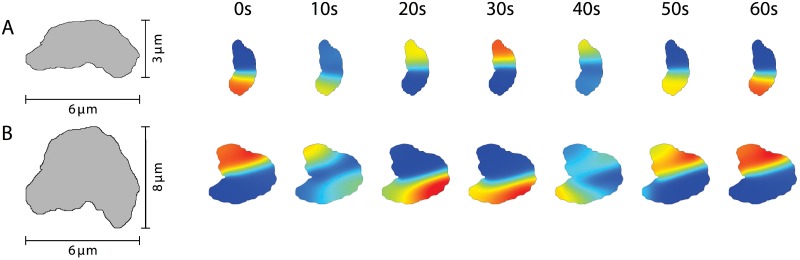
Oscillation patterns found in irregularly shaped cells. A) Crescent shaped irregular geometry. B) Irregular shaped geometry. Results from calculations in 3d. Both geometries have a height of 0.4 *μ*m. Parameter values as in Table 1. The maximal grid size used in the computations was 0.38 *μ*m (A) and 0.7 *μ*m (B).

In addition to regular formed cell bodies, stable Min oscillations have also been observed in cells whose shape is highly divergent from the basic geometric shapes discussed earlier [28]. One of the geometries presented there is a crescent shaped cell with an irregular boundary and a height of 0.4 *μ*m as seen in Fig 10A. As long as the irregularity of the shape is small enough, the pattern is essentially the same as for the rod-shaped geometry, Fig 10A and S18 Movie, which is similar to the experimental pattern [28] and in line with previous calculations [53]. For larger deviations from the wild-type geometry, the proteins can also get localized in sufficiently large “crypts” of the boundary, Fig 10B and S19 Movie. The patterns in these irregular shapes are thus a natural extension of the patterns in the branched geometries presented in Fig 9.

## Summary, discussion, and conclusion

In this work, we presented a computational study of Min-protein self-organization. We analyzed the dynamic equations introduced in Ref. [29] that had already been shown to reproduce the patterns observed in normally shaped bacteria as well as on flat surfaces. Here, we showed that these equations also reproduce the patterns on patchy flat and topographically structured surfaces. This refutes the contrary statement that was made in Ref. [33]. In addition, we showed that the dynamic equations have solutions corresponding to patterns that have been reported in deformed bacteria. These results significantly enlarge the number of experimentally observed Min-protein patterns that are captured by the mechanism studied here. Although other published mechanisms might have a similar power, we are not aware of studies explicitly showing this.

Nevertheless, the dynamic equations presented above cannot be the last word on the Min-protein dynamics. Indeed, several significant issues remain. First of all, rather dramatic parameter changes are necessary for obtaining the patterns in live bacteria and in vitro. Although, different parameter sets might require less dramatic differences between these two cases. They might also lead to better quantitative agreement between the experimental patterns and the solutions to the dynamic equations. In particular, they could lead to a steeper increase in the MinE density towards the trailing end as shown by the experimental distributions. However, we do not think that looking for other parameter values is the way to go. First, because we lack sufficient experimental constraints on the parameter values. Second and more importantly, there is a qualitative difference between the traveling wave patterns *in vitro* and the solutions to the dynamic equations, namely the residence time *τ*_*D*_ of MinD (and *τ*_*E*_ of MinE) on the membrane. Within the mean-field equations studied in the present work, they can be estimated to be

$$\begin{array}{c} \langle \tau_{D}\rangle ={\left(\omega_{E}c_{E}+\omega_{ed}c_{e}\right)}^{-1}+{\left(\omega_{de,c}+\omega_{de,m}\right)}^{-1} \end{array}$$ (8)

$$\begin{array}{c} \left\langle \tau_{E}\right\rangle =\frac{2(\omega_{de,m}+\omega_{ed}c_{d}+\omega_{e})}{\omega_{de,c}\omega_{ed}c_{d}+(\omega_{de,c}+\omega_{de,m})\omega_{e}}. \end{array}$$ (9)

The residence times vary with space as they depend on the MinD and MinE densities. From Eq 8 we infer that *τ*_*D*_ decreases with increasing MinE density. Consequently, it decreases towards the trailing edge of a wave. This is common to all mechanisms in which the detachment rate of MinD depends only on the density of MinE, but is opposite to what has been observed experimentally [22]. From this discrepancy between the theory and the experiments, we conclude that an important qualitative feature of the MinD-MinE-membrane interaction is missing. One possibility is that membrane-bound MinD mutually stabilize their membrane-bound state. In fact, the co-operative binding term, Eqs (3) and (6), already suggests such a stabilizing effect of MinD interactions. Early experimental evidence for membrane-bound MinD clusters was given by Hu et al [36].

In spite of recent advances made on the molecular nature of the dynamic MinD-MinE interactions [42], we clearly see a need for further experiments revealing the mechanism of MinD binding to the membrane and the MinE-induced unbinding of MinD from the membrane. In addition to the cooperative binding used in the present work and which had been introduced by Huang et al. [19], other possibilities exist. Notably, binding of MinD to the membrane through a first order process and subsequent formation of clusters could be possible. It has been studied theoretically in some earlier works [18, 37, 38]. In addition to addressing these qualitative features, it will be, in the future, of particular interest to obtain a quantitative picture of the Min-protein dynamics. In particular, they should include the experimental determination of the number of MinD and MinE molecules in individual bacteria. Such measurements would allow for a comparison of the experimental and theoretical phase-space toplogies, which should give valuable information on the mechanism underlying Min-protein pattern formation.

Still, we also see room for more theory at the current stage. First of all and quite generally, the impact of molecular noise on self-organized patterns is not well understood. This holds in particular for patterns that are formed through molecules attaching from a bulk phase to surfaces. A generic mechanism for protein accumulation at one extremity of a rod-shaped bacterium has been studied with respect to the frequency of stochastic switching between the two possible states in the case of small fluctuations [52]. It will be interesting to extend such investigations to the case of stochastic switching of the Min system [47, 52] and to stuttering, which refers to the phenomenon of skipping a transition event in the standard pole-to-pole oscillations [54]. It is noteworthy that, in both cases, fluctuations decrease with increasing system size and thus molecule number. Finally, we also see an interesting theoretical avenue in studying energetic and evolutionary aspects of the Min-system.

## Supporting information

S1 Movie Illustration of the finite element grid used for the numerical solution of the dynamic equations for a rectangular geometry. The maximal mesh size specified for Comsol was 1.33 *μ*m.
(AVI)

S2 Movie Illustration of the finite element grid used for the numerical solution of the dynamic equations for a rectangular geometry. The maximal mesh size specified for Comsol was 0.14 *μ*m.
(AVI)

S3 Movie Min-protein dynamics on an L-shaped patch. Min surface-waves guided along an L-shaped patch of width 40 *μ*m corresponding to Fig 5.
(AVI)

S4 Movie Min-protein dynamics on an L-shaped patch. Min surface-waves guided along an L-shaped patch of width 25 *μ*m corresponding to Fig 5C.
(AVI)

S5 Movie Min-protein dynamics on an L-shaped patch. Min surface-waves guided along an L-shaped patch of width 60 *μ*m corresponding to Fig 5C.
(AVI)

S6 Movie Min-protein dynamics on a topographically structured surface. Min surface-waves are guided by sufficiently deep grooves on the surface. Data corresponding to Fig 6A and 6B.
(AVI)

S7 Movie Min-protein dynamics in a rectangular shaped body. Pole-to-pole oscillation in a rectangular shaped body of size 3 *μ*m × 5 *μ*m × 1.1 *μ*m corresponding to Figs 7 and 8A.
(AVI)

S8 Movie Min-protein dynamics in a square shaped body. Standing wave along the diagonal of a square shaped body with side length 3 *μ*m and height 1.1 *μ*m corresponding to Figs 7 and 8C.
(AVI)

S9 Movie Min-protein dynamics in a circular shaped body. Standing wave in a circular shaped body with a diameter of 5 *μ*m and height 1.1 *μ*m corresponding to Fig 7.
(AVI)

S10 Movie Min-protein dynamics in a triangular shaped body. Standing wave in a triangular shaped geometry with side length 5 *μ*m and height 1.1 *μ*m corresponding to Fig 7.
(AVI)

S11 Movie Min-protein dynamics in a rectangular shaped body. Standing wave with two nodes in a rectangular shaped body of size 3 *μ*m × 5 *μ*m × 1.1 *μ*m corresponding to Fig 8A.
(AVI)

S12 Movie Min-protein dynamics in a rectangular shaped body. Standing wave with two nodes along the diagonal of a rectangular shaped body of size 5 *μ*m × 8 *μ*m × 1.1 *μ*m corresponding to Fig 8B.
(AVI)

S13 Movie Min-protein dynamics in a rectangular shaped body. Standing wave along the short axis of a rectangular shaped body of size 5 *μ*m × 8 *μ*m × 1.1 *μ*m corresponding to Fig 8B.
(AVI)

S14 Movie Min-protein dynamics in a square shaped body. Wave travelling along the perimeter of a square shaped side length 3 *μ*m and height 1.1 *μ*m corresponding to Fig 8C.
(AVI)

S15 Movie Min-protein dynamics in a branched cell. Rotating wave pattern for branches of lengths 2.4 *μ*m, 2.7 *μ*m, and 3.0 *μ*m corresponding to Fig 9A.
(AVI)

S16 Movie Min-protein dynamics in a branched cell. Symmetric standing wave pattern for branches of lengths 3.0 *μ*m, 1.1 *μ*m, and 1.0 *μ*m corresponding to Fig 9B.
(AVI)

S17 Movie Min-protein dynamics in a branched cell. Oscillation between two poles for branches of lengths 1.8 *μ*m, 1.9 *μ*m, and 0.5 *μ*m corresponding to Fig 9C.
(AVI)

S18 Movie Min-protein dynamic in an irregular cell. Small crescent shaped irregular geometry corresponding to Fig 10A.
(AVI)

S19 Movie Min-protein dynamic in an irregular cell. Large crescent shaped irregular geometry corresponding to Fig 10B.
(AVI)

S1 Appendix Equations in two dimensions. Contains the dynamic equation corresponding to Eqs (1)–(7) in a two-dimensional geometry.
(PDF)
