# Supplementary material for: Effects of geometry and topography on Min-protein dynamics
Source: PLoS One. 2018 Aug 30;13(8):e0203050. doi: 10.1371/journal.pone.0203050 (PMC6117030; doi:10.1371/journal.pone.0203050)
Supplement: S1 Appendix — Contains the dynamic equation corresponding to Eqs (1)–(7) in a two-dimensional geometry. (PDF) [file pone.0203050.s020.pdf]

Effects of Geometry and Topography on Min-Protein Dynamics - SI

Lukas Wettmann[1], Mike Bonny[1], Karsten Kruse[1,2,*],

[1]Theoretische Physik, Universität des Saarlandes, Saarbrücken, Germany
[2]NCCR Chemical Biology, Departments of Biochemistry and Theoretical Physics, University of Geneva, Geneva, Switzerland

S1 Appendix. Equations in two dimensions. The distributions of MinD and MinE in the buffer are given by the densities c_D and c_E. The distributions of MinD, MinDE-complexes, and MinE on the membrane are, respectively, denoted by c_d, c_{de}, and c_e. The dynamic equations read

$$\partial_t c_D = D_D \Delta c_D - c_D(\omega_D + \omega_{dD} c_d)(c_{\max} - c_d - c_{de})/c_{\max} + (\omega_{de,m} + \omega_{de,c})c_{de} \quad (1)$$

$$\partial_t c_E = D_E \Delta c_E - \omega_E c_E c_d + \omega_{de,c} c_{de} + \omega_e c_e \quad (2)$$

$$\partial_t c_d = D_d \Delta c_d + c_D(\omega_D + \omega_{dD} c_d)(c_{\max} - c_d - c_{de})/c_{\max} - \omega_E c_E c_d - \omega_{ed} c_e c_d \quad (3)$$

$$\partial_t c_{de} = D_{de} \Delta c_{de} + \omega_E c_E c_d + \omega_{ed} c_e c_d - (\omega_{de,m} + \omega_{de,c})c_{de} \quad (4)$$

$$\partial_t c_e = D_e \Delta c_e + \omega_{de,m} c_{de} - \omega_{ed} c_e c_d - \omega_e c_e. \quad (5)$$

Here, Δ denotes the Laplace operator in two dimensions.
